# Towards Operational Modeling and Forecasting of the Iberian Shelves Ecosystem

**DOI:** 10.1371/journal.pone.0037343

**Published:** 2012-05-29

**Authors:** Martinho Marta-Almeida, Rosa Reboreda, Carlos Rocha, Jesus Dubert, Rita Nolasco, Nuno Cordeiro, Tiago Luna, Alfredo Rocha, João D. Lencart e Silva, Henrique Queiroga, Alvaro Peliz, Manuel Ruiz-Villarreal

**Affiliations:** 1 CESAM-Dep Física, Universidade de Aveiro, Aveiro, Portugal; 2 Instituto do Ambiente e Desenvolvimento, Universidade de Aveiro, Aveiro, Portugal; 3 CESAM-Dep Biologia, Universidade de Aveiro, Aveiro, Portugal; 4 Centro de Oceanografia, Universidade de Lisboa, Lisboa, Portugal; 5 Instituto Español de Oceanografía, Centro Oceanográfico A Coruña, Galicia, Spain; University of Oxford, United Kingdom

## Abstract

There is a growing interest on physical and biogeochemical oceanic hindcasts and forecasts from a wide range of users and businesses. In this contribution we present an operational biogeochemical forecast system for the Portuguese and Galician oceanographic regions, where atmospheric, hydrodynamic and biogeochemical variables are integrated. The ocean model ROMS, with a horizontal resolution of 3 km, is forced by the atmospheric model WRF and includes a Nutrients-Phytoplankton-Zooplankton-Detritus biogeochemical module (NPZD). In addition to oceanographic variables, the system predicts the concentration of nitrate, phytoplankton, zooplankton and detritus (mmol N m^−3^). Model results are compared against radar currents and remote sensed SST and chlorophyll. Quantitative skill assessment during a summer upwelling period shows that our modelling system adequately represents the surface circulation over the shelf including the observed spatial variability and trends of temperature and chlorophyll concentration. Additionally, the skill assessment also shows some deficiencies like the overestimation of upwelling circulation and consequently, of the duration and intensity of the phytoplankton blooms. These and other departures from the observations are discussed, their origins identified and future improvements suggested. The forecast system is the first of its kind in the region and provides free online distribution of model input and output, as well as comparisons of model results with satellite imagery for qualitative operational assessment of model skill.

## Introduction

Providing operational oceanographic data on biological and chemical variables has become an issue of concern over the last years. The demand for this kind of information arises from a range of fields and applications such as scientific research on marine ecosystems, monitoring of seawater quality and decision-making support for marine and coastal management. A recent questionnaire conducted by ICES-WGOOFE (International Council for the Exploration of the Sea, Working Group on Operational Oceanographic Products for Fisheries and Environment) showed that temperature, currents, salinity, chlorophyll standing stock and primary production were the most requested products among ocean sciences scientific community, who scored several biological parameters in the top 10 rankings of products on demand [1]. There is a well known increasing interest in combined physical, chemical and biological operational products, including near-real time and forecast, which are currently possible due to facilitated access to computational resources, development of numerical solutions and improvement of modelling algorithms and performance.

The marine policies implemented in many countries to protect the sea from increasing environmental pressures has urged the need for monitoring systems of seawater quality. In the European Union, the Water Framework Directive and the Marine Strategy Framework Directive launched in 2000 and 2008, commit the member states to the evaluation and monitoring of the ecological status of their river basins/coastal waters and marine waters, respectively (e.g., [2], [3]). The setting up of the European Earth Monitoring Programme GMES (Global Monitoring for Environment and Security) by the European Commission, which includes an important ocean component and aims to be fully operational by 2014, highlights the interest on reliable and up-to-date marine environmental information [4].

Biogeochemical models constitute a valuable tool for operational oceanography when coupled with circulation models. They can complement the time and space limitation of observations and offer the possibility to help explain processes and variability. The simplest version of these models are the NPZD (Nutrients-Phytoplankton-Zooplankton-Detritus) models. They can give information on concentration of the biological state variables over time, and have strong potentialities for analysis and prediction. Nevertheless, modelling the ocean biogeochemical properties has a number of difficulties that make it a challenging task. The parameters used in biogeochemical models are often not well constrained in the literature and finding an appropriate set of parameters through trial runs is seldom straightforward. In addition, independently of the level of complexity of a biogeochemical model, it is still a great simplification of the reality.

The strong dependency of biology on hydrodynamics requires an ocean circulation model able to reproduce adequately the main features and variability in the modelling domain, at both seasonal and event time scales. In regional coastal domains, sufficiently high resolution is required to resolve the shelf mesoscale eddy processes, which depends upon high performance computational facilities.

Operational modelling products of ocean biogeochemical variables are rarely provided by online operational systems, presumable because of the difficulties mentioned above. In the western Iberia, an early experience of chlorophyll forecast in 1998 in the Gulf of Cadiz is referred in Pinard and Woods [5], Chap 11. The GMES ocean data server MyOcean (http://www.myocean.eu) provides modeled biogeochemical data for the European northwest shelf since April 2011, including the northernmost part of the Iberian maritime region. The model used has been implemented operationally over the last years in the seas around the UK and Ireland [6]. The Project EasyCO (model products in several European maritime regions, including the western Iberia) foresees to distribute biogeochemical data, although this type of information is currently available only for the western French coast. Other regions in Europe with operational modelling products for biology are the Baltic Sea, developed by the Baltic ocean community [7], and the Mediterranean Sea, developed by the Istituto Nazionale di Oceanografia e di Geofisica Sperimentale, OGS, in collaboration with other Italian Institutions (INGV, CINECA and GOS-ISAC-CNR) [8], [9]. Both available through MyOcean.

In this contribution we present an operational biogeochemical forecast system implemented for the Atlantic Iberian shelf ecosystem: Portuguese and Galician shelf and slope and nearby oceanic regions. This product is the first of its kind in the region and provides free online access to all ocean state and biogeochemical simulated variables. The hydrodynamic forecast system is working since 1^st^ November 2008 and the biogeochemical module is available operationally since 10^th^ June 2011.

The next section describes the ocean model setup, the atmospheric model providing surface data for the ocean model, the biogeochemical model and the operational system setup. Then, results are presented and compared with observations. In the last section we present the summary and outlook of upcoming improvements.

## Models

### Ocean Model

The numerical model implemented is the Regional Ocean Modelling System, ROMS [10], [11], [12]. ROMS is a free-surface terrain-following primitive equation hydrostatic model with Boussinesq approximations and configurable for realistic applications. ROMS has been used in a variety of time and space scales, from very small regions, like harbours, to the coastal, large and global scale.

The model configuration in use has been successfully applied in the western Iberian region during roughly the last ten years, to many different studies, like dispersion of larvae [13], [14], [15], river plumes [16], [17], [18], [19] and pollution transport [20], [21]. The Gibraltar exchange flow and the large scale circulation associated with the Azores Current are included in the modelling configuration and were based on [22], [23].

**Figure 1:**
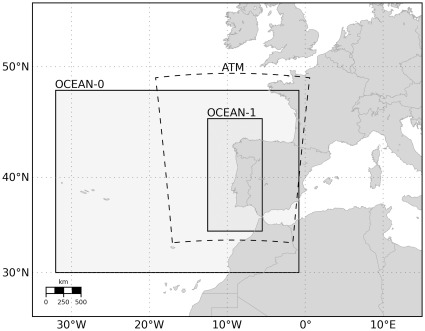
Study region with nested grids configuration of the atmospheric model (ATM) and ocean model (OCEAN-0 and target domain OCEAN-1).

The ocean model uses two different domains, illustrated in Figure 1 as OCEAN-0 and OCEAN-1. The large domain, OCEAN-0, has horizontal resolution of 1/10°, able to resolve the large scale circulation features, and vertical resolution of 30 s-levels with enhanced surface and bottom resolution. The simulations of OCEAN-0 started from rest with temperature and salinity climatologies from [24] and [25], used to initialise and constrain the open boundaries of this domain. The surface was forced with fluxes from the Atlas of Surface Marine Data [26]. Monthly geostrophic velocities (referenced to 1200 m) and Ekman velocities were calculated from the climatologies and applied along the lateral boundaries. The Mediterranean inflow/outflow and spreading was parametrised following [22]. This configuration ran for several years until a stable kinetic solution was attained by year five. The monthly mean of this last year is considered a good resolution climatology and thus used to provide initial and boundary data to the high resolution configuration OCEAN-1.

**Figure 2:**
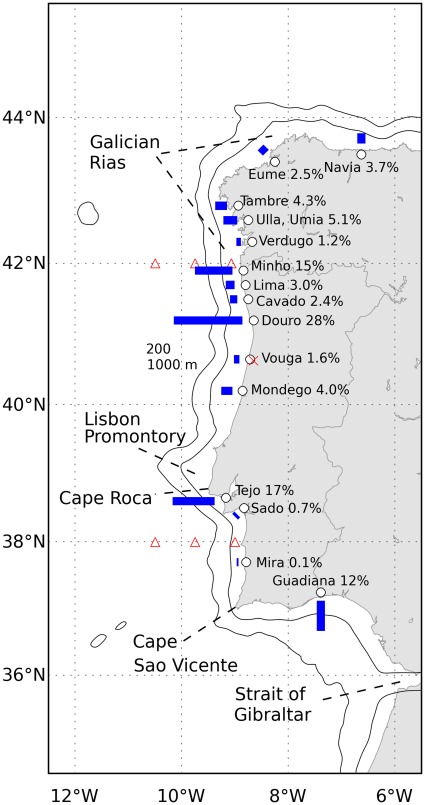
Operational ocean model domain with the location of geographical features and landmarks referenced through the text. The model grid covers the Portuguese and Galician coasts. The main riverine sources of the model are indicated together with the corresponding percentages of fresh water inputs (rivers were introduced as monthly climatologies). The three triangles at the latitudes 42°N and 38°N indicate the locations of surface currents (42°only), temperature and chlorophyll comparisons, between model and observations, done in this work. The cross near the Vouga river mouth is the location of wind comparison.

The target domain OCEAN-1 (Figures 1 and 2) has a horizontal resolution of about 3 km extending from Gibraltar to North Galicia (∼1200 km). The domain width is about 600 km. In the vertical 60 s-levels are used with increased near bottom resolution, in order to deal with the Mediterranean exchange (parametrised in the same way as in OCEAN-0). This model was initialised at 1^st^ November 2008 and forced with realistic high resolution surface fluxes from a local solution of the atmospheric model WRF (described in the next section).

The main riverine fresh water inputs from Portugal and Galicia were included in both domains as monthly climatologies. All rivers used are indicated in Figure 2 together with the corresponding percentage of freshwater input in the model domain. The river discharges were obtained from the Portuguese Water Institute (INAG, http://www.inag.pt) and from estimations presented in [27]. A fixed value of 12°C was used for river temperatures.

### Atmospheric Model

The model which provides surface forcing data to the ocean domain OCEAN-1 (Figure 2), is an operational implementation of the Weather Research and Forecasting Advance Research model (WRF-ARW) version 3.2.1 [28]. The WRF model is driven by GFS forecasts [29] with an horizontal resolution of 0.5° x 0.5°, and vertical domain extending from a surface pressure of 1000 hPa to 0.27 hPa, discretized in 64 vertical unequally-spaced levels (15 levels are located below 800 hPa and 24 levels above 100 hPa).

The atmospheric forecasts are performed daily by the Group of Meteorology and Climatology of Aveiro University (CliM@UA, http://climetua.fis.ua.pt) and encompass the Portuguese mainland, Madeira and Azores archipelagos. The oceanic operational system uses the weather forecasts for Portuguese mainland domain with spatial resolution of 25 km (Figure 1).

The atmospheric model in use by CliM@UA was configured after a set of numerical experiments described by Ferreira [30]. Model outputs were tested against observations and the best combination of model parametrisations was found for the region.

### Biological Model

The biogeochemical model contains a 4-component nitrogen based ecosystem NPZD model (based on [31]), computing 4 state variables: nutrients (nitrate), phytoplankton, zooplankton and detritus, all expressed in mmol N m^−3^. Chlorophyll *a* (mg m^−3^) is derived from phytoplankton concentration using a variable chlorophyll:C ratio (*θ*) that is a function of light and nutrients availability, and a C:N ratio of 6.625 (mmol C/mmol N), i.e., a Redfield ratio. The equation for *θ* describes the proportion of photosynthetically fixed carbon that is used for chlorophyll a biosynthesis considering the model of Geider et al. [32], which implementation in the ocean model is described in Gruber et al. [33]. The 3D time evolution of the concentration of any of the biogeochemical variables (*B_i_*) follows the general equation:
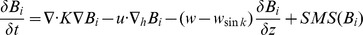
where the terms in the right hand side account for diffusion, horizontal advection, vertical mixing and sink minus source (*SMS*) biological processes, respectively. *K* is the eddy kinematic diffusivity tensor, ***u*** is the horizontal velocity of the fluid, *w* and *w_sink_* are the vertical velocity of the fluid and the vertical sinking rate of the biogeochemical tracer, respectively, with the exception of zooplankton and nitrate, to which no sinking rate is attributed.

The biogeochemical processes included in *SMS* are specific for each variable and represent the following conceptual description: phytoplankton uptakes nitrate at a rate that is dependent on the instantaneous nitrate concentration; temperature and light intensity/PAR (photosynthetically available radiation). PAR at the surface is calculated as 43% of the incident radiation and attenuated with depth as it is absorbed by water and chlorophyll. Phytoplankton dies at a constant linear rate, been automatically incorporated to the detritus pool. Zooplankton growth relies on its grazing on phytoplankton, which rate depends on prey concentration, and its assimilation efficiency. A constant excretion rate is attributed to zooplankton, providing a source of nutrients to the nitrate pool. Zooplankton incorporates to the detritus pool at a constant linear mortality rate. Mineralization of detritus is formulated as direct transformation to nitrate at a constant nitrification rate. Model parameters for the sink/source terms selected to represent our region of study are listed in Table 1. A detailed description of the biogeochemical model equations, including sink-source terms, can be found in Koné et al. [34].

**Table 1:** Parameter values of the NPZD model.

Parameter	Value	Units
Light attenuation in seawater	0.04	m^−1^
Light attenuation by chlorophyll	0.024	m^−1^ (mg Chla m^−3^)^−1^
Initial slope of the P-I curve	1.0	mg C (mg Chla W m^−2^ d)^−1^
C:N ratio for phytoplankton	6.625	mol C (mol N)^−1^
Maximum Cellular chlorophyll:C ratio	0.03	mg Chla (mg C)^–1^
Half-saturation for phytoplankton NO_3_ uptake	1.5	mmol N m^−3^
Zooplankton half-saturation constant for ingestion	1.0	mmol N m^−3^
Maximum zooplankton growth rate	0.9	d^–1^
Zooplankton assimilation coefficient	0.75	n.d.
Phytoplankton mortality (to detritus) rate	0.03	d^–1^
Zooplankton mortality (to detritus) rate	0.1	d^–1^
Zooplankton specific excretion rate	0.1	d^–1^
Detrital mineralisation to NO_3_ rate	0.05	d^–1^
Sinking velocity for phytoplankton	0.5	m d^–1^
Sinking velocity for detritus	5	m d^–1^

The concentration of nitrate and chlorophyll *a* for the model initial and boundary conditions were supplied by the climatological data sets World Ocean Atlas 2005 [35] and SeaWiFS, respectively. The initial and boundary data of phytoplankton and zooplankton were derived from chlorophyll *a*. Detritus were introduced constant with the value 0.02 mmol N m^−3^. Boundary conditions were supplied seasonally. The riverine inputs of nitrate and chlorophyll were used constant along the year and were obtained from Ferreira et al. [36] and from the European Environment Agency (http://www.eea.europa.eu). The values used are listed in Table 2.

**Table 2:** River inputs of nitrate and chlorophyll.

River	Nitrate (µg l^−1^)	Chlorophyll (µg l^−1^)
Navia	0.1	0.1
Eume	6.4	0.1
Tambre	23.2	0.1
Ulla, Ulmia	13.3	0.1
Verdugo	4.4	0.1
Minho	34.7	2.0
Lima	11.8	2.4
Cavado	33.2	0.9
Douro	88.5	5.4
Vouga	44.4	0.1
Mondego	2.3	5.0
Tejo	21.0	8.5
Sado	21.3	9.6
Mira	10.6	0.9
Guadiana	5.2	0.1

The biological model runs as a module integrated in the ocean model. It became operational at 10^th^ June 2011.

### Operational System

The ocean and biological modelling system was implemented operationally with OOFε (Operational Ocean Forecast Python Engine, [37]). OOFε is a comprehensive set of tools, written in the Python programming language, which creates and operates the model input/output and controls all the required tasks for the operationality of the ocean model. The operational engine executes daily analysis and forecast (currently three days), whose cycles are repeated continuously in a robust and fully automated manner. The operational engine also includes a visualisation module able to produce many types of slices and plots from the ocean model inputs/outputs. Some graphical outputs of the current implementation can be seen at the homepage of the forecast system, http://neptuno.fis.ua.pt/oof. Among the several outputs are horizontal slices at several depths of temperature, salinity and chlorophyll, as well as forcing wind, sea surface height, currents, etc. For a quick comparison to observational data sets, the web site also shows satellite observations, namely sea surface temperature from O&SI-SAF (Ocean & Sea Ice Satellite Application Facility, www.osi-saf.org) and from OSTIA (Operational Sea Surface Temperature and Sea Ice Analysis, http://ghrsst-pp.metoffice.com), and high resolution chlorophyll processed and distributed by IFREMER (http://cersat.ifremer.fr/science/ocean color).

The full model data sets are freely available through the Python OPenDAP server Pydap (http://pydap.org). OPeNDAP is an efficient protocol for allowing the remote access of data sets (e.g [38], [39]). Data usually available as a set of different individual large data files is made available in an efficient and consistent way by OPeNDAP servers.

The system implemented with OOFε has been successfully operating the ROMS model in other locations, like the Brazilian region [40] and the Northern Gulf of Mexico [41].

## Results

The results presented give an overview of model ability to reproduce the hydrodynamic (surface currents and surface temperature) and chlorophyll fields for the four months following the operational implementation of the biogeochemical model (10^th^ June 2011 to 10^th^ October 2011). These preliminary results are thus addressed as a general reliability assessment rather than a thorough validation, which would need more comparisons with observations and for a longer time period. The period studied corresponds to summer and early autumn, which is characterised in the western coastal region by recurrent upwelling conditions. Therefore, the model is evaluated during one of the most dynamic and biologically productive seasons of the year.

Surface model currents are compared with a high frequency radar data, owned by the Spanish Merchant Navy, at three locations off Vigo (latitude 42°N and longitudes 9°W, 9.75°W and 10.5°W). Given the importance of atmospheric forcing for the ocean model to adequately reproduce the circulation, the surface wind provided by the WRF atmospheric model is compared with wind measurements at University of Aveiro weather station. Then, in order to assess model ability to reproduce coastal blooms, the onset and evolution of a strong upwelling/coastal bloom event (13^th^ to 26^th^ of July) is shown, comparing modeled sea surface temperature and chlorophyll concentration fields in the model with satellite observations (OSISAF-EUMETSAT Ocean & Sea Ice Satellite Application Facility and IFREMER optimised interpolation of MODIS, SeaWiFS and MERIS observations, respectively). To better evaluate the model skill for surface temperature and chlorophyll along the four months, and at different locations within the domain, time series comparing model results and satellite data are presented for two latitudes (42°N and 38°N) in three locations for each latitude, corresponding to middle shelf, off the shelf break and open-sea (same longitudes as used for currents comparison at 42°N). Finally, the model skill in the whole domain is further explored by plotting model bias of SST and chlorophyll which allow to evaluate whether the model is over-or underestimating the observations.

Due to the lack of reliable observational data other than satellite chlorophyll and SST for the time period under study, we do not further explore model skill assessment methods [42], [43]. For the same reason, neither concentration of nitrate or the vertical structure of the variables was analysed in the current study.

### Surface Wind and Currents

**Figure 3:**
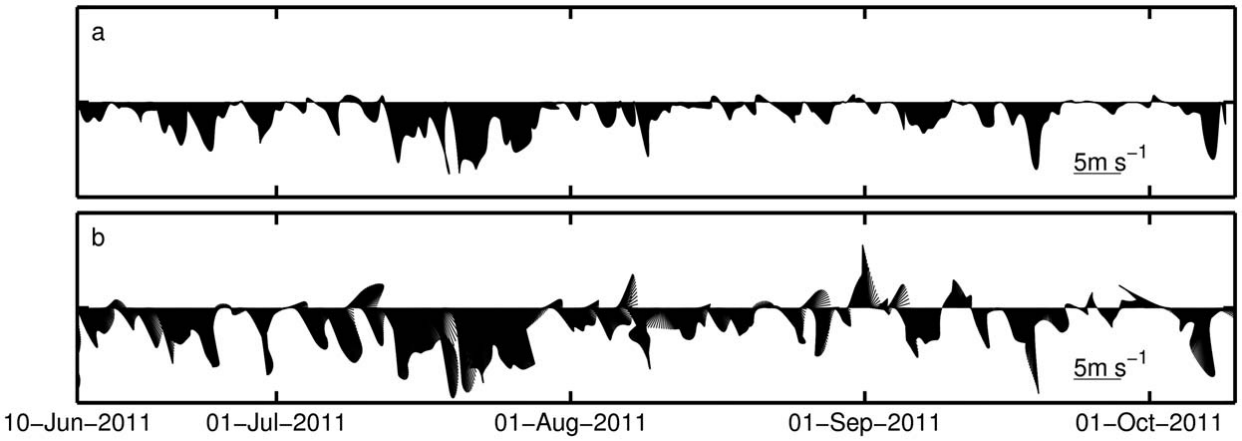
Wind measured by the Aveiro University automatic weather station (a) and ocean model forcing wind at the same location (b). The site is indicated in Figure 2 as x close to the Vouga river mouth.

The surface wind measured at Aveiro University weather station (the location is indicated in Figure 2 as x, near the Vouga river mouth) is displayed in Figure 3A. The wind field, upwelling favourable, is typical of the summer season for Western Iberian region, with predominance of northerly winds [44]. The most intense event is observed between 13 and 28 of July, with wind intensity higher than 5 m s^–1^ during most of the days. Figure 3B shows the ocean model forcing wind, generated by the WRF atmospheric model, interpolated at the same location. In general, the modeled wind is more intense than the observed one and the wind inversion after the relaxation is also more prominent.

**Figure 4:**
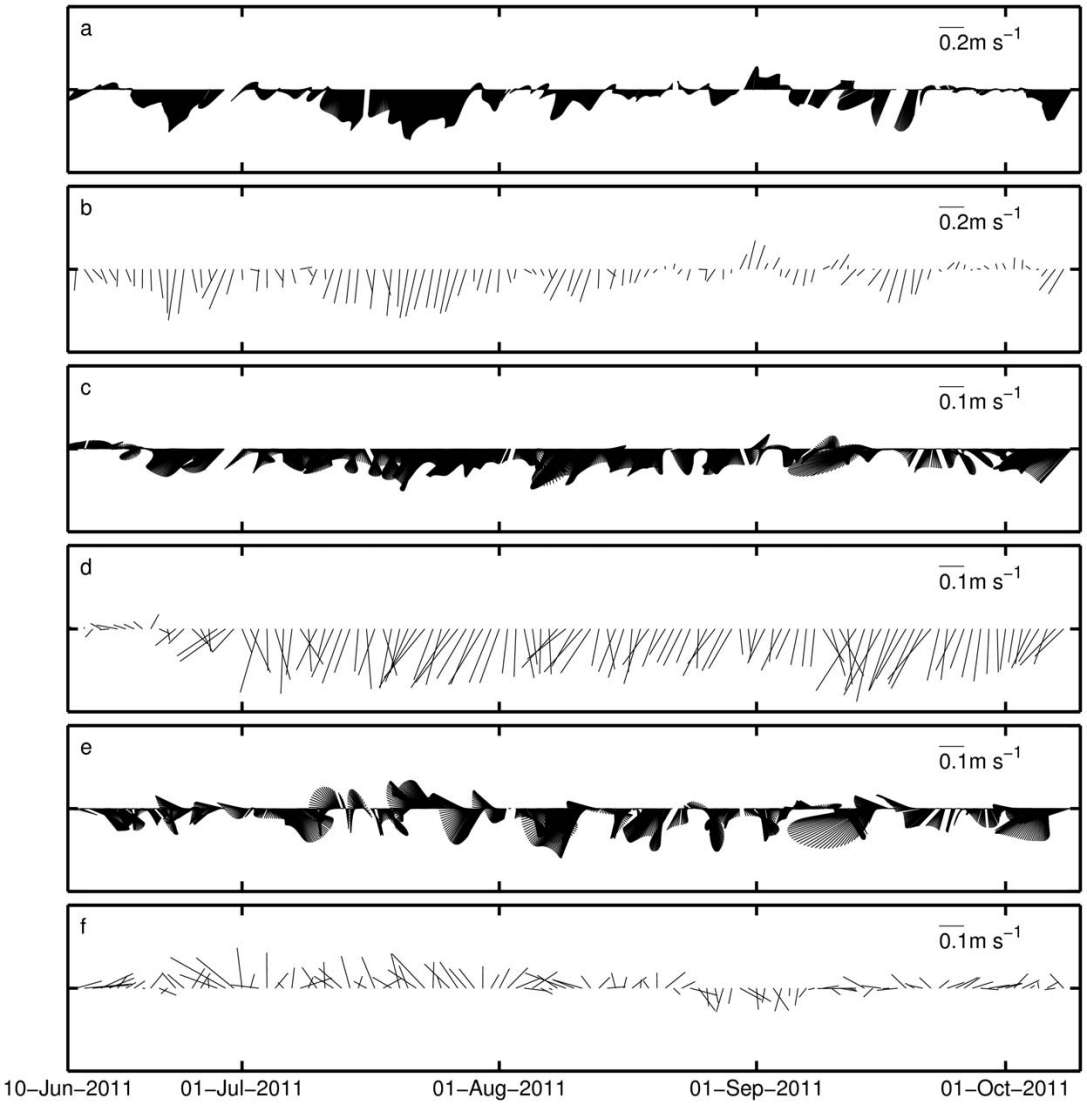
Observed and modeled surface currents at three locations along the latitude 42°N at the longitudes 9°W (a and b), 9.75°W (c and d) and 10.5°W (e and f). These locations are shown in Figure 2 as triangles.

The comparison of modeled and observed surface currents at the latitude 42°N is shown in Figure 4. (the three comparison sites are indicated in Figure 2). The data shown has been filtered with a low-pass filter with sub-diurnal cutoff frequency. In the shelf (Figure 4A, 4B), the model reproduces correctly the intensity and direction of the current field, which is dominated by an equatorward flow in response to the upwelling favourable wind forcing, with presence of typical velocities of 0.2 m s^−1^, and maximum values of about 0.35 m s^–1^ associated to the peak of wind forcing by day 22^nd^ of July. In the vicinity of the shelf break (Figure 4C, 4D), the southward tendency is also present in the observations and model results, but the model presents in general higher intensities. In the open-ocean (Figure 4E, 4F) the mesoscale variability is very high and the model cannot reproduce the observed eddy activity.

### Coastal Bloom Event, 13^th^ to 26^th^ July 2011

The general trend on chlorophyll concentration simulated along the considered period was that expected for a typical summer situation in the Iberian upwelling region. Successive coastal blooms appeared associated with upwelling pulses, and were more persistent in the northwest coast around Galicia and in the proximity of Cape Roca. In several occasions the bloom occupied the coast from North to South.

**Figure 5:**
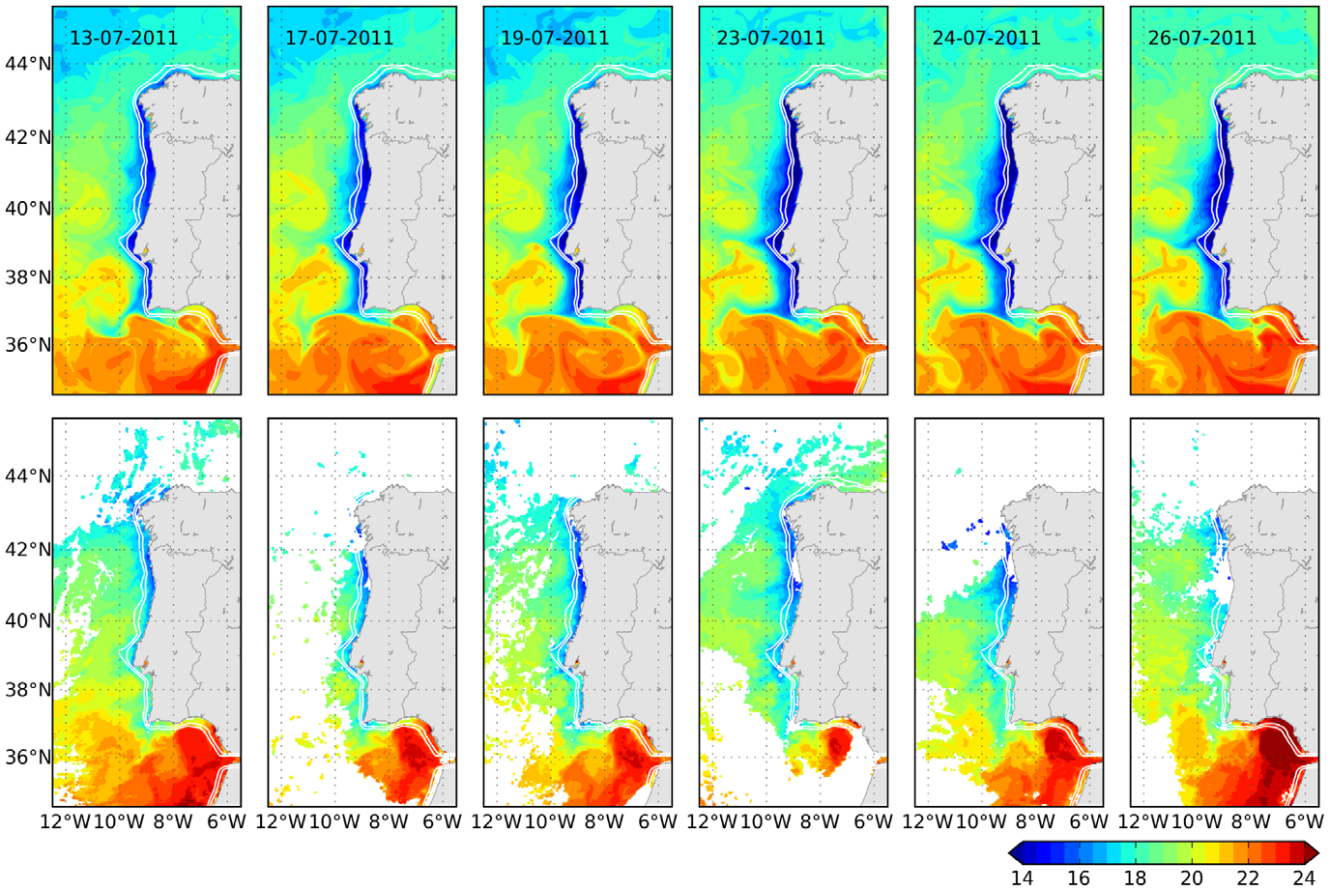
Sea surface temperature from model (upper panel) and from satellite observations provided by OSISAF (lower panel, processed by Meteo-France/CMS-Lannion in the framework of the OSISAF project). Snapshots between 13^th^ and 26^th^ July 2011 are depicted, illustrating one episode of upwelling intensification and coastal bloom (next Figure).

**Figure 6:**
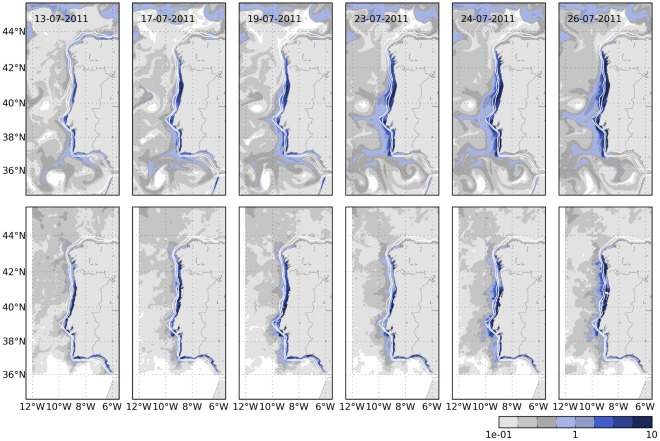
Same as previous figure but showing the chlorophyll coastal bloom. Chlorophyll observations are provided by IFREMER and obtained from OC5 optimised interpolation of MODIS, SeaWiFS and MERIS observations.

We selected the period 13^th^ to 26^th^ July, characterised by strong northerly wind, upwelling favourable, that led to the onset and evolution of a conspicuous coastal bloom, to evaluate model performance reproducing this kind of blooms. Surface temperature and chlorophyll in the model were compared with satellite observations (Figures 5 and 6). Surface temperature shows a distinctive band of cold, upwelled water near the coast which was narrower and more restricted to the North coast in the satellite images than in the model. Two filaments with origin in Cape Roca and Cape S. Vicente appeared in the model, and some evidence of their presence was also observable in the satellite (e.g. days 23^rd^ and 24^th^). As for the phytoplankton bloom, the increase in chlorophyll concentration along the coast (up to about 6 mg m^−3^) appeared at about the same time in the model and in the satellite, around 13^th^ of July. The trend was particularly well reproduced in the region between 40°N and 42°N, where the concentration reached 10 mg m^−3^, whereas in the southwest coast (37°N to 39°N), the concentration in the model was higher than in observations (highest values around 6 and 2 mg m^−3^, respectively). In contrast, the model was not able to reproduce the high inner shelf chlorophyll concentration along the South coast of Portugal and the Gulf of Cadiz (36°N to 37°N). The bloom situation continued on the next days, spreading offshore with increasing chlorophyll concentration. By day 23^th^, the bloom seemed to decrease on satellite observations, and then it intensified again (24^th^ to 26^th^). This decrease was not observed in the model and the bloom continued extending offshore and along the coast. As a consequence, at the end of July, the bloom was more intense and spread farther offshore in the model than in the satellite observations. This is probably related with a slower relaxation of the upwelling winds in the model than in the real ocean.

### SST and Chlorophyll Time Series

**Figure 7:**
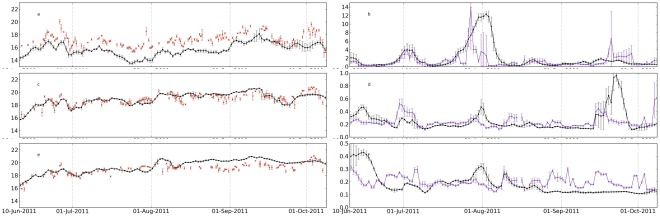
Observed and modeled (in black) sea surface temperature (a, c and e) and chlorophyll (b, d and f) at latitude 42°N and longitudes 9°W, 9.75°W and 10.5°W (locations indicated as triangles in Figure 2. The satellite observations are distributed by OSISAF and by IFREMER. Note the different vertical scales for chlorophyll. Each data points is the average of the data inside a circle of radius 7 km. Error bars are the corresponding standard deviations.

**Figure 8:**
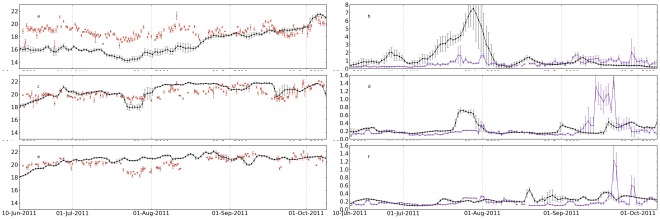
Same as **Figure 7** but for the latitude 38 °**N.**

The model skill for sea surface temperature and chlorophyll was investigated comparing time series between the model and the satellite at six points within the domain (Figures 7 and 8). The comparison sites are indicated in Figure 2 and represent different zones: middle shelf; offshore vicinity of shelf brake; and offshore zone, for two latitudes, in the northern and southern part of the domain (42°N and 38°N). At both latitudes, the modeled shelf surface temperature was lower than temperature from satellite during most part of the analysed period, with typical bias higher than 1°C (Figure 7A and Figure 8A). At the offshore sites the model had a better skill reproducing both the low and high (event scale) variability of the surface temperature (Figure 7C, 7E and Figure 8C, 8E). The relatively colder waters near the coast may be associated with several factors including over-mixing and inability of the model to re-stratify after strong wind events, deficient parametrisation of air-sea heat fluxes and wind-stress. However, the mixing parametrisations we have used are the ones that are standard and recommended in the majority of ROMS applications. The K-Profile Parameterization, KPP model, described in detail in Large et al. [45], is fully tested for most of the mesoscale applications. It can be argued that near the coast other mixing schemes could perform better, but they usually require additional local information. On the other hand, the colder bias near the coast have been reported in several upwelling systems studies, and attributed to the lack of resolution of the wind stress field near the coast, in which the wind curl is not properly resolved (e.g., [46], [47]). A recent comparison across different upwelling systems [48] shows that the bias is common to all upwelling systems and that in part there is a warm tendency of SST data bases.

The surface chlorophyll comparisons show that the relatively simple biological model is able to reproduce many of the observed features, mainly in terms of variability. At 42°N the model simulated the main episodes of chlorophyll concentration variability, namely the shelf blooms by the end of July and beginning of August (Figure 7B). Chlorophyll concentration in both peaks was similar in the model and in satellite observations. The model peak in August lasted longer, most probably as a consequence of the model difficulty to represent the relaxation of the upwelling (Figure 3). A third bloom appeared in early autumn (end of September) that was not captured by the model in the shelf (Figure 7B), but it was captured next to the shelf brake (Figure 7D). In general, chlorophyll concentration in the model was of the same order as satellite values, except for the most offshore location (10.5°W) at the end of the period, when model values tended to be systematically lower (Figure 7F).

For the latitude 38°N (Figure 8) the model also shows some sensitivity to the event at the end of July, but giving much higher chlorophyll concentrations than the observed ones. The model simulated blooms in the shelf (Figure 8B) and near the shelf brake (Figure 8D) that were considerably higher than in the satellite observations (or even non-existent in the satellite). This behaviour is believed to be a consequence of the stronger modeled upwelling in the southern coast, which is reflected in lower temperatures with a bias of near 2°C at the shelf until middle of August (Figure 8A). It is worth noting that the southern shelf is considerably narrower than the northern shelf (Figure 2), which may play a role in this difference due to the lower numerical discretization of the shelf in this region, and hence higher sensitivity to wind forcing accuracy. Conversely, the chlorophyll peaks observed offshore in early autumn were not properly captured in the model (Figure 8D 8F). Still, the model shows evidences of being able to reproduce the slowly varying base concentration, around 0.2 mg m^−3^.

### SST and Chlorophyll Model Bias

**Figure 9:**
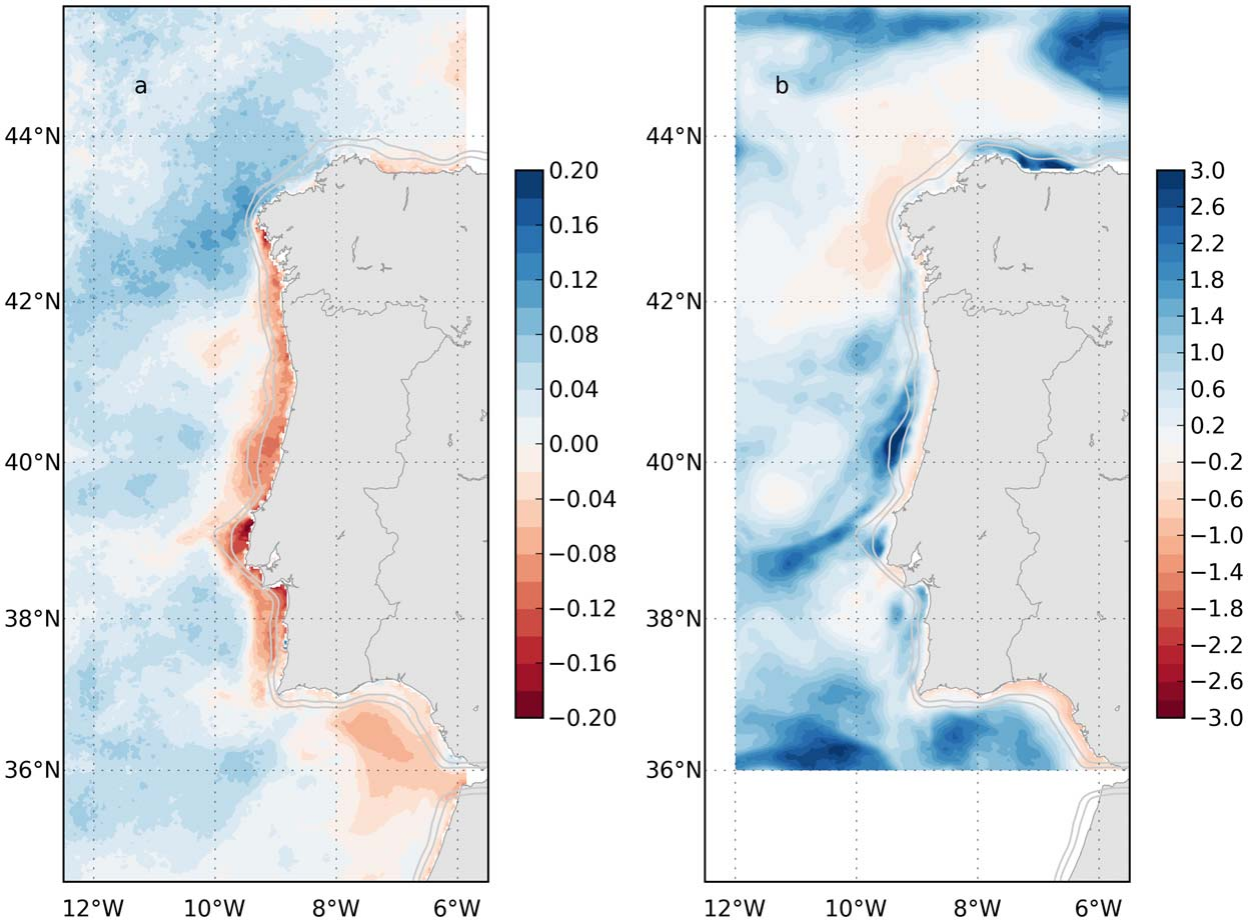
Model bias of sea surface temperature (a) and chlorophyll (b) for the period 10^th^ June 2011 to 10^th^ October 2011.

The model bias (MB) indicates whether the model is overestimating or underestimating the observations, being the model results as better as MB is closest to zero. For each grid point, it was calculated as:
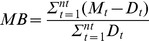
where *M_t_* and *D_t_* are the modeled and observed values at time index *t*, respectively. Figure 9 shows the model bias of sea surface temperature and chlorophyll for the domain. There is a systematic negative bias of temperature over the western shelf, as expected, because of the higher intensity of modeled wind than measured (Figure 3), leading to higher upwelling and lower surface temperatures extending offshore, beyond the shelf break. The bias of chlorophyll was lowest in the inner shelf and the offshore region around Galicia. Higher differences are found throughout the western outer shelf and in general near the model domain boundaries. In some regions, like the shelf off Asturias (northern coast) and the outer shelf and slope around 40.5°N, modeled chlorophyll concentration was three times higher than satellite values. The chlorophyll bias in the outer shelf is supposed to be related with the negative bias in temperature, i.e., higher upwelling. Near the boundaries, the differences reflect the usage of climatological boundary conditions biogeochemical variables, which vary very slowly and are very often in disagreement with reality.

## Discussion

A ROMS based operational hydrodynamic system was implemented covering the Portuguese and Galician coast. The ocean model has high horizontal and vertical resolution (about 3 km and 60 s-levels) and is running since November 2008. It uses monthly climatological lateral boundaries provided by a climatological run of a larger scale model with about 10 km of horizontal resolution (see Figure 1). The surface is forced with realistic momentum, temperature and salinity fluxes modeled by a local implementation of the atmospheric model WRF. The main rivers of the region are included in the model as monthly climatological means. Also included is the Peliz et al. [22] Gibraltar inflow/outflow parametrisation.

The operational system is totally automatic and runs systematically daily analysis and three days forecasts. This implementations relies on the OOFε (Operational Ocean Forecast Python Engine) described in [37].

More recently, on 10^th^ June 2011, a 4-component nitrogen based biogeochemical model was initiated operational as a module of the ocean model. The rivers are considered a source of nitrates and chlorophyll with input concentrations constant along the year.

The modeled ocean and biological state variables of the first four months of the coupled hydrodynamic and biogeochemical model were compared with observations for validation. The observations consisted in radar measured surface currents, wind from a weather station and satellite sea surface temperature and chlorophyll.

The model reproduced adequately the wind-forced circulation over the shelf, typical of the western Iberia summer season. Namely the surface currents, the broad spatial and temporal variability of the upwelling circulation and consequent sea surface temperature. However, the consistent temperature deficit, reaching 2°C is an indication of overestimated upwelling. This arises from the difficulty of the atmospheric model to properly resolve the wind field near the coast. Such overestimation leads to artificial surplus of nutrients within the euphotic layer, and consequent overestimation of the intensity and duration of the phytoplankton bloom. It is worthy to note that the model has no relaxation of sea surface temperature, a numerical resource frequently used in long term simulations, to assure convergence to the correct seasonal pattern.

In the northern shelf the model was able to simulate the main blooms observed by the satellite along the upwelling season, and gave similar chlorophyll concentrations, although showing differences in the duration of the most intense coastal bloom. On the other hand, model results in the southern shelf tended to overestimate observed blooms. The main differences between model and satellite have hydrodynamic origin, namely the more intense upwelling and slower wind relaxation in the model. Nevertheless, in general, the base concentration and variability of surface chlorophyll is reasonably reproduced by the NPZD model. It is important to note that satellite observations can neither be taken as the “real value” in the ocean, since they have associated errors.

A more thorough validation of the model would require further comparisons of model data with high quality in situ measurements (buoys and sampling stations), which are not easily available. Comparisons for a longer time period are also needed. Though, work is underway to cope with the limitations of the current validation.

By providing information about the model skill, the comparison with observations gives clues about possible steps to improve the operational system. Among the future developments, the most important for the coastal and shelf regions, is to have a better representation of the wind field. The local WRF solutions should be improved, namely through optimisation of modelling parametrisations and data assimilation. Ensemble forecasts and assimilation of observations are currently in phase of test and implementation. The rivers have great impact on the shelf dynamics and the use of climatological monthly discharges is certainly an important limitation of the model. Another important limitation in the rivers is the use of constant concentration of nutrients (although the total amount of nutrients varies with river flux). Attempts to estimate at least seasonal patterns should be addressed. This can be done with ensemble Kalman filter optimisation techniques, for instance. The complexity of the biogeochemical model can also be increased to include more variables that may improve model results. Currently tests are underway adding a second subgroup of nitrates (ammonia) and subdividing the detritus in small and large size.

Because of the use of climatological lateral boundaries, the model lacks the ability to reproduce the eddy mesoscale features away from the continental shelf. Some operational eddy-resolving global models with data assimilation are available (like HYCOM, http://www.hycom.org, and MERCATOR http://www.mercator-ocean.fr) and our regional model can be offline nested in the nowcasts and forecasts of such models (e.g., [49]). Also modifying boundary information for the biogeochemical variables, by using data from a global of large-scale parent model, may improve the model results offshore. The results obtained so far with the operational oceanic and biogeochemical implementation are satisfactory and encourage the continuation of the work. The limitations detected incentive improvement efforts, which are currently being studied and tested. In the meantime, results of the oceanic and biogeochemical models are already available online at the web page of the operational system, http://neptuno.fis.ua.pt/oof. Also available are some satellite observations, for comparison. The full model input/output data sets are free to be accessed by any end user through OPenDAP, at the same web address.

## References

[1] Berx B, Dickey-Collas M, Skogen MD, Roeck YHD, Klein H (2011). Does operational oceanography address the needs of fisheries and applied environmental scientists?. Oceanography.

[2] Borja A (2005). The European water framework directive: A challenge for nearshore, coastal and continental shelf research.. Cont Shelf Res.

[3] Borja A, Elliott M, Carstensen J, Heiskanen AS, van de Bund W (2010). Marine management - Towards an integrated implementation of the European Marine Strategy Framework and the Water Framework Directives.. Mar Pollut Bull.

[4] Brachet G (2004). From initial ideas to a European plan: GMES as an exemplar of European space strategy.. Space Policy.

[5] Pinardi N, Woods J (2002). Ocean forecasting: conceptual basis and applications..

[6] Siddorn JR, Allen JI, Blackford JC, Gilbert FJ, Holt JT (2007). Modelling the hydrodynamics and ecosystem of the North-West European continental shelf for operational oceanography.. J Mar Syst.

[7] Neumann T (2000). Towards a 3D-ecosystem model of the Baltic Sea.. J Mar Syst.

[8] Lazzari P, Teruzzi A, Salon S, Campagna S, Calonaci C (2010). Pre-operational short-term forecasts for the Mediterranean Sea biogeochemistry.. Ocean Sci.

[9] Teruzzi A, Salon S, Bolzon G, Lazzari P, Campagna S (2011). Operational forecasts of the biogeochemical state of Mediterranean Sea.. Mercator ocean Newsletter.

[10] Shchepetkin AF, McWilliams JC (2005). The Regional Ocean Modeling System (ROMS): A split-explicit, free-surface, topography-following coordinates ocean model.. Ocean Model.

[11] Penven P, Debreu L, Marchesiello P, McWilliams JC (2006). Evaluation and application of the ROMS 1-way embedding procedure to the central california upwelling system.. Ocean Model.

[12] Haidvogel DB, Arango H, Budgell WP, Cornuelle BD, Curchitser E (2008). Ocean forecasting in terrain-following coordinates: Formulation and skill assessment of the Regional Ocean Modeling System.. J Comput Phys.

[13] Marta-Almeida M, Dubert J, Peliz A, Queiroga H (2006). Influence of vertical migration pattern on retention of crab larvae in a seasonal upwelling system.. Mar Ecol Prog Ser.

[14] Marta-Almeida M, Dubert J, Peliz A, dos Santos A, Queiroga H (2008). A modelling study of the Norway Lobster (*Nephrops norvegicus*) larval dispersal in southern Portugal: predictions of larval wastage and self-recruitment in the Algarve stock.. Can J Fish Aquat Sci.

[15] Peliz A, Marchesiello P, Dubert J, Marta-Almeida M, Roy C (2007). A study of crab larvae dispersal on the Western Iberian Shelf: Physical processes.. J Mar Syst.

[16] Silva PA, Ramos M, Marta-Almeida M, Dubert J (2007). Water Exchange Mechanisms Between Ria de Aveiro and the Atlantic Ocean.. J Coastal Res.

[17] Otero P, Ruiz-Villarreal M, Peliz A (2008). Variability of river plumes off Northwest Iberia in response to wind events.. J Mar Syst.

[18] Otero P, Ruiz-Villarreal M, Peliz A (2009). River plume fronts off NW Iberia from satellite observations and model data.. ICES J Mar Sci.

[19] Otero P, García-García MRVL, Marta-Almeida M, Cobas M, González-Nuevo G (2011). Walking on the sea side: Modeling and observational efforts of the Iberian Margin Ocean Observatory (RAIA).. OCEANS 2011.

[20] Ruiz-Villarreal M, González-Pola C, Otero P, Días del Río G, Lavín A (2006). Circulation in the Galicia-Southern Bay of Biscay: Reanalysis of the circulation influencing the Prestige oil spill. In: European Operational Oceanography: present and future.. EuroGOOS,.

[21] Sotillo MG, Fanjul EA, Castanedo S, Abascal AJ, Menendez J (2008). Towards an operational system for oil-spill forecast over Spanish waters: Initial developments and implementation test.. Mar Pollut Bull.

[22] Peliz A, Dubert J, Marchesiello P, Teles-Machado A (2007). Surface circulation in the Gulf of Cadiz: Model and mean flow structure.. J Geophys Res.

[23] Peliz A, Marchesiello P, Santos AMP, Dubert J, Teles-Machado A (2009). Surface circulation in the Gulf of Cadiz: 2. Inflow-outflow coupling and the Gulf of Cadiz slope current.. J Geophys Res.

[24] Levitus S, Boyer TP (1994). World ocean atlas 1994 volume 4: Temperature..

[25] Levitus S, Burgett R, Boyer TP (1994). World ocean atlas 1994 volume 3: Salinity..

[26] da Silva A, Young AC, Levitus S (1994). Atlas of surface marine data 1994, volume 1: Algorithms and procedures..

[27] Otero P, Ruiz-Villarreal M, Peliz A, Cabanas JM (2010). Climatology and reconstruction of runoff time series in northwest Iberia: influence in the shelf buoyancy budget off Ria de Vigo.. Sci Mar.

[28] Skamarock WC, Klemp JB, Dudhia J, Gill DO, Barker DM (2008). A Description of the Advanced Research WRF Version 3..

[29] NOAA (2003). The GFS Atmospheric Model..

[30] Ferreira AP (2007). Sensibilidade às parametrizações físicas do WRF nas previsões à superfície em Portugal Continental..

[31] Fasham MJR, Ducklow HW, McKelvie SM (1990). A nitrogen-based model of plankton dynamics in the oceanic mixed layer.. J Mar Res.

[32] Geider RJ, MacIntyre HL, Kana TM (1997). Dynamic model of phytoplankton growth and acclimation: responses of the balanced growth rate and the chlorophyll a:carbon ratio to light, nutrient-limitation and temperature.. Mar Ecol Prog Ser.

[33] Gruber N, Frenzel H, Doney SC, Marchesiello P, McWilliams JC (2006). Eddy-resolving simulation of plankton ecosystem dynamics in the California Current System.. Deep Sea Res Part I.

[34] Koné V, Machu E, Penven P, Andersen V, Garçon V (2005). Modeling the primary and secondary productions of the southern Benguela upwelling system: A comparative study through two biogeochemical models.. Global Biogeochem Cy.

[35] Garcia HE, Locarnini RA, Boyer TP, Antonov JI (2006). World Ocean Atlas 2005, Volume 4: Nutrients (phosphate, nitrate, silicate).. Washington, D.C.: NOAA Atlas NESDIS.

[36] Ferreira JG, Simas T, Nobre A, Silva MC, Schifferegger K (2003). Identification of Sensitive Areas and Vulnerable Zones In Transitional and Coastal Portuguese Systems..

[37] Marta-Almeida M, Ruiz-Villarreal M, Otero P, Cobas M, Peliz A (2011). OOFε: A Python engine for automating regional and coastal ocean forecasts.. Environ Modell Softw.

[38] Cornillon P, Adams J, Blumenthal MB, Chassignet E, Davis E (2009). NVODS and the Development of OPeNDAP.. Oceanography.

[39] Signell RP, Carniel S, Chiggiato J, Janekovic I, Pullen J (2008). Collaboration tools and techniques for large model datasets.. J Mar Syst.

[40] Marta-Almeida M, Pereira J, Cirano M (2011). Development of a pilot Brazilian operational ocean forecast system, REMO-OOF.. J Oper Ocean.

[41] Zhang X, Marta-Almeida M, Hetland RD (2012). A high-resolution pre-operational forecast model of circulation on the Texas-Louisiana continental shelf and slope.. J Oper Ocean.

[42] Allen JI, Holt JT, Blackford J, Proctor R (2007). Error quantification of a high-resolution coupled hydrodynamic-ecosystem coastal-ocean model: Part2. Chlorophyll-a, nutrients and SPM.. J Mar Syst.

[43] Jolliff JK, Kindle JC, Shulman I, Penta B, Friedrichs MAM (2009). Summary diagrams for coupled hydrodynamic-ecosystem modelskillassessment.. J Mar Syst.

[44] Wooster WS, Bakun A, Mclain DR (1976). The seasonal upwelling cycle along the eastern boundary of the north atlantic.. J Mar Res.

[45] Large WG, McWilliams JC, Doney SC (1994). Oceanic vertical mixing: a review and a model with a nonlocal boundary-layer parameterization.. Rev Geophys.

[46] Capet X, Marchesiello PP, McWilliams JC (2004). Upwelling response to coastal wind profiles.. Geophys Res Lett.

[47] Otero P, Ruiz-Villarreal M (2008). Wind forcing of the coastal circulation off north and northwest Iberia: Comparison of atmospheric models.. J Geophys Res.

[48] Dufois F, Penven P, Whittle CP, Veitch J (2012). On the warm near shore bias in Pathfinder monthly SST products over Eastern Boundary Upwelling Systems.. Ocean Model.

[49] Barth A, Alvera-Azcárate A, Weisberg RH (2008). Benefit of nesting a regional model into a large-scale ocean model instead of climatology: Application to the West Florida Shelf.. Cont Shelf Res.

